# Wear analysis of slideway in emulsion pumps based on finite element method

**DOI:** 10.1038/s41598-024-51943-6

**Published:** 2024-01-22

**Authors:** Dalong Wang, Ran Li, Hao Liu, Jian Ye

**Affiliations:** 1 School of Mechanical and Electrical Engineering, China University of Mining and Technology Beijing, Beijing, 100083 China; 2 Beijing Tianma Intelligent Control Technology Co., Ltd., Beijing, 101399 China; 3 Graduate School, China Coal Research Institute, Beijing, 100013 China

**Keywords:** Engineering, Mechanical engineering

## Abstract

Wear is a common issue in the operation of emulsion pumps. When it becomes severe, it can lead to machine downtime and economic losses. This paper aims to investigate the wear phenomenon on the slideway of emulsion pumps using Archard’s wear model and the finite element method. The fretting friction and wear experiment was used to calibrate the parameters of the numerical model. Based on the established numerical model, a parametric analysis is conducted on the slideway experiencing the most severe wear. It is observed that the wear amount initially increases and then decreases as the crankshaft speed increases. Furthermore, a smaller clearance between the slide and the slider results in a reduced wear amount on the slideway. This study presents an effective numerical simulation method for studying the wear of emulsion pumps.

## Introduction

Coal is the lifeblood of China’s national economy, and its consumption has accounted for more than half of China’s energy consumption for many years^1,2^. The emulsion pump station is the power equipment of the hydraulic support in the coal mining working face. The safe and stable operation of the pump is of great significance to energy saving and orderly production^3,4^. As a necessary guarantee for the safe and efficient operation of coal mining, the emulsion pump station is required to ensure a stable supply of fluid to the coal mining working face^5,6^. It is mainly composed of an emulsion tank, emulsion pump set, and hydraulic control system. The power end of the pump set provides power to the emulsion pump set, including the crankcase, crankshaft, slider, slideway, bearings, and shaft tiles. With the development of coal mining technology in China, the demand for large hydraulic supports is becoming more and more significant, which requires the flow rate of the emulsion pumping station to be continuously increased. The increase in flow rate leads to an increase in the force on the parts at the power end and an increase in the contact force on the friction pair, so the working conditions of the key friction pair at the power end become harsher. The harsh working condition makes the friction and wear problem between the slideway and the slider become prominent. Once a large wear occurs, it may cause the output pressure of the pumping station to be unstable, and may lead to the shutdown of the mining face. Therefore, it is necessary to establish an appropriate model to study the friction and wear behavior on the slide and slideway.

Various forms of wear cause about 1/3–1/2 of the world’s energy waste, and the main reason for mechanical equipment failure is wear due to friction, so researchers have been very concerned about the friction and wear phenomenon in mechanisms. Yousefi simulated the tool wear phenomenon in punching operation in LS-DYNA^7^. Agrawal studied the interaction between the tool and the rock during the boring process of the tunnel boring machine using EDEM software^8^. Lian studied the surface fatigue life and wear behavior of multi-wheeled roadway rails and found that wear increases when the slip rate rises^9^. Oksanen has studied the wear behavior in rolling-slip contact of ductile iron and wire rope in the laboratory environment and use conditions^10^. At present, there are few studies on friction and wear phenomena in emulsion pumping stations, and in the available papers, researchers usually analyze simplified friction and wear processes by building theoretical models. Experiment is the most reliable way to study friction behavior^11^, however, conducting real machine wear experiments on an emulsion pump station can be very expensive, and there is a need to find a less expensive way to predict wear, the finite element method can be used to study friction and wear more conveniently and intuitively.

There is a lot of literature on the simulation of wear using the finite element method. In Wang’s work, he studied the shearing process of solid particles between two parallel plates using the coupled finite element and discrete element method and found that the strong collision between particles at low pressure increased the overall friction coefficient^12^. Woldman, et al., proposed a model to predict the wear of mechanical structures in sandy environment based on the finite element method, compared with the experimental results, the model has achieved good simulation results^13^. Jin used the finite element method to establish a wear prediction model for rails considering contact stress and material hardness, and the effectiveness of the wear prediction method was verified by the actual measured values of the actual rails^14^. Arjmandi studied the effects of weaving methods of fabric components on the wear rate and structural reliability of hydrogel composites under compressive load were studied by finite element method^15^. Rezaei used the adaptive finite element method to study the wear process of the radial sliding bearing in contact with the rotating shaft and found that the wear depth is directly related to the gap size^16^. Wu used the finite element method to study the thermal elastic–plastic deformation and residual stress after the wheel slides on the rail and found that the frictional thermal load of the rail has a significant effect on both^17^.

Scholars have proposed many models that can be used to describe wear, and the classical Archard model is still the most commonly used way to predict the wear behavior of materials^18^. Based on the Archard model, Yu proposed a quasi-dynamic method to evaluate the characteristics of ball bearings, it is found that the wear life of the bearing increases approximately linearly with the increase of the inner ring groove curvature^19^. Hu used the Archard model to study the three-dimensional metal turning process of ordinary ceramic tools and ultrafine-grained tools and found that the wear depth of ordinary ceramic tools is several times that of ultrafine-grained tools^20^. Telliskivi studied the famous disk-to-disk dry wear based on the Archard model, the simulation results are in good agreement with the experimental results^21^. Bose uses the differential form of Archard’s wear law to obtain the wear depth at the contact node^22^. Reichelt used the Archard model to describe the relationship between wear volume and sliding distance and normal force when performing wear experiments on 100Cr6 material samples^23^. Babu established the finite element model of the stamping process and studied the wear problem under different die material combinations based on the Archard model^24^.

To ensure the safe and stable operation of the emulsion pump station, in this paper, the friction and wear behavior of emulsion pump station on coal mine working surface is studied. For the slider and slideway mechanism in the power end, even if the emulsion pump station is in normal operation, the speed of the reciprocating friction pair is very low near the stop point, and it is not easy to establish the oil film, so the solid contact is difficult to avoid. Therefore, the reciprocating friction pair in the power end is often the most serious position of wear. This paper calculates the development trend of wear based on Archard wear model. The fretting friction and wear experiment was used to calibrate the parameters of the numerical model. The effects of different rotational speeds and clearances on friction and wear in motion pairs are studied using the finite element model. The conclusion can help to reduce friction and wear in the design of emulsion pumps.

## Materials and methods

### Experimental materials

**Figure 1:**
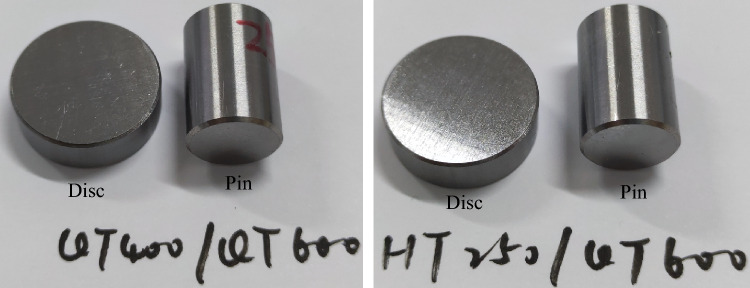
Partial samples in fretting friction and wear test.

In order to verify the accuracy of the simulation results, the fretting friction and wear experiments on different material combinations were conducted. The materials used include QT400, QT500, QT600, and HT250. The disc is a cylinder with a diameter of 24 mm and a height of 7.88 mm. The pin is a cylinder with a diameter of 15 mm and a height of 22 mm. Some of them are shown in Fig. 1. The disc and pin were first rough-polished with sandpaper of different particle sizes such as 600 mesh and 800 mesh, and then the test surface was polished to the *Ra* is about 0.02 μm. The polished sample was immersed in acetone and ultrasonically cleaned. The verticality of the disc and pin should be less than 0.005 mm. The material combinations in the experiments are shown in Table 1.

**Table 1:** Material combinations in fretting friction and wear experiments.

Combinations	Pin	Disc
1	QT600	QT400
2	QT600	QT500
3	HT250	HT250
4	QT600	HT250
5	QT500	QT500

### Fretting friction and wear experiment

**Figure 2:**
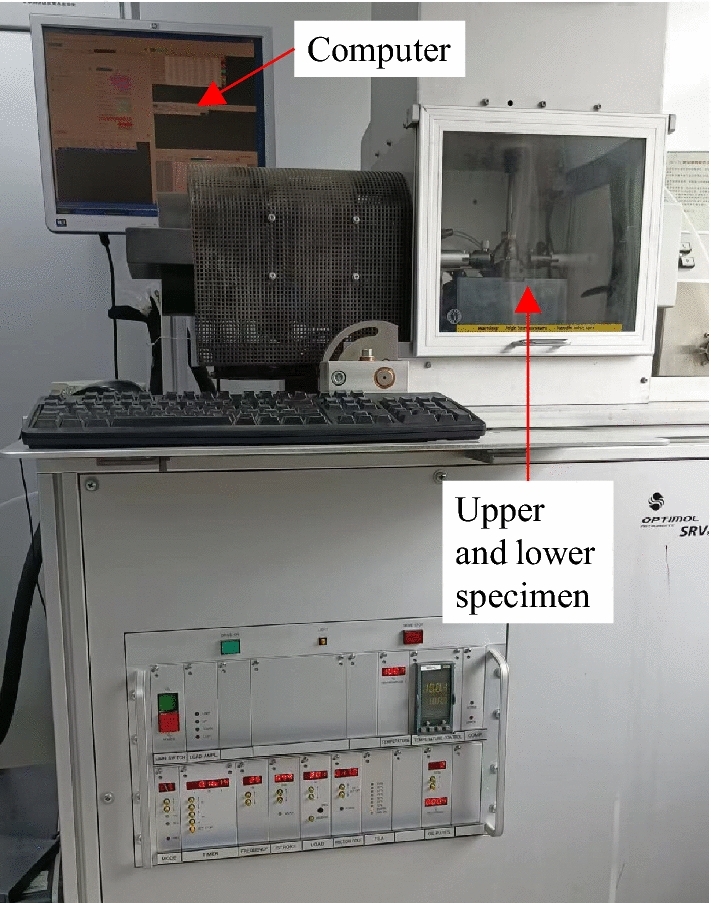
Fretting friction and wear test device.

The fretting friction and wear experiments were carried out on the SRV-IV fretting friction and wear tester of Optimol Company, Germany. The experimental device is shown in Fig. 2. The contact mode of the friction pair is plane/plane contact. During the experiment, the disc was fixed, the pin was reciprocating linear motion, and the reciprocating friction and wear mode were adopted under the condition of no lubrication. The friction tester can directly give the dynamic friction coefficient curve. The experimental temperature was 22 °C, the relative humidity was 30%, the normal loads were 100 N and 30 N, the experimental period was one hour, the frequency was 50 Hz, and the stroke was 1 mm. In order to remove the impurities attached to the surface, the disc and pin were ultrasonically cleaned in acetone solution for 10 min before each experiment. More detailed information about the experiment can be found in reference^25^.

### Experimental results

After the fretting friction and wear experiment was completed, the sample was immersed in an acetone solution for ultrasonic cleaning. After drying, the surface morphology of the wear scar was observed using a non-contact three-dimensional surface profiler, and then the details of the scratch surface were observed using a Scanning Electron Microscope (SEM).

The friction coefficient *μ* is the ratio of the *F*_*f*_ and *F*_*N*_:

1 $$\mu = \frac{{F_{f} }}{{F_{N} }},$$

where *F*_*f*_ is the maximum friction; *F*_*N*_ is the pressure acting on the contact surface.

**Figure 3:**
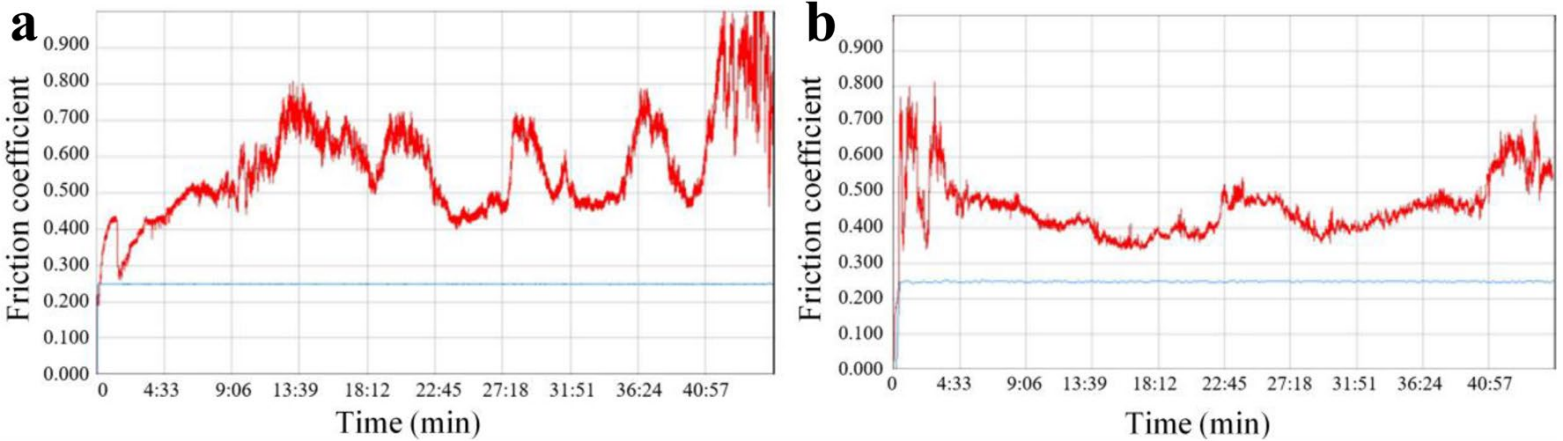
Friction coefficient curves of QT600(Pin)/HT250(Disc) under different loads. (**a**) 30 N. (**b**) 100 N.

The variation curve of the friction coefficient with the number of cycles can reflect the actual wear intensity during the whole fretting friction and wear experiment. Figure 3 shows the variation curve of friction coefficient *μ* with time under different loads. It can be seen from the Fig. 3 that the curve at a load of 30 N is divided into two stages. In stage I, the friction coefficient rises steadily and the change range is very small. In stage II, the friction coefficient changes dramatically, and the fluctuation range is significantly higher than in the previous stage. The fretting wear at a load of 100 N is mainly divided into two stages: Stage I, in the beginning, the contact between the surfaces of the friction pair did not reach a stable state, and the friction coefficient had undergone a wide range of changes with a large frequency of fluctuation. Stage II, with the increase of friction distance, when the surface contact of the friction pair becomes stable, the friction coefficient decreases to a minimum and then increases, the overall change frequency of the friction coefficient decreases, the amplitude decreases, and gradually approaches a stable state. It is worth noting that in the stable stage of friction coefficient, the friction coefficient decreases obviously with the increase of load.

**Figure 4:**
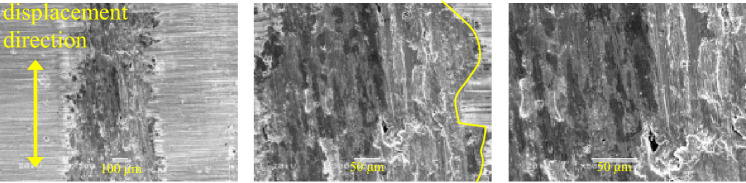
SEM images of fretting wear surface of QT600/QT400 combination.

Wear surface morphology can intuitively reflect the friction and wear situation, taking QT600/QT400 combination as an example, Fig. 4 shows the surface morphology of three different scales of wear scar of the specimen with material QT600 under a load of 30 N. The Fig. 4 shows a typical adhesive zone and abrasive wear, it can be observed that a large number of wear debris accumulates on both sides of the wear scar along the displacement direction and form a bulge. Under the action of repeated shear stress of fretting wear, there are a large number of pits formed after the wear debris falls off in the edge area of the wear mark. A part of the wear debris is squeezed at the edge of the wear scar to cause plastic deformation and form a flat extrusion layer, and another part of the wear debris is brought into the central area of the wear scar by the reciprocating action of the specimen and is continued to grind to produce secondary fretting wear. Finally, a dense wear debris area is formed in the central area of the wear scar.

**Figure 5:**
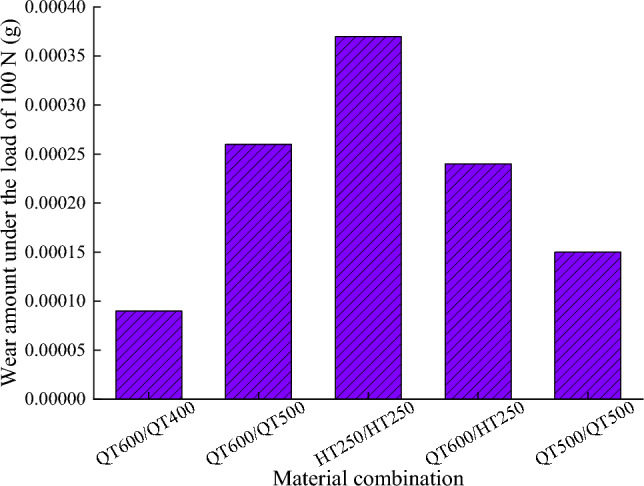
Fretting wear under different material combinations.

Figure 5 shows the wear amount of different material combinations under the load of 100 N, it can be seen from the Fig. 5 that HT250 with the lowest elastic modulus and hardness has the largest wear amount, but when the specimen combination is QT600/QT500, the wear amount is also large. The high hardness of the two object materials of the friction pair does not necessarily reduce the friction and wear.

## Numerical simulation of power end running state

The microscopic appearance of different materials after wear was studied through experiments, and the amount of material wear was obtained. In order to observe the dynamic change of the wear state of the slideway during the operation of the emulsion pump more comprehensively, a finite element model was established in this section.

### The finite element model

#### Establishment of the model

**Figure 6:**
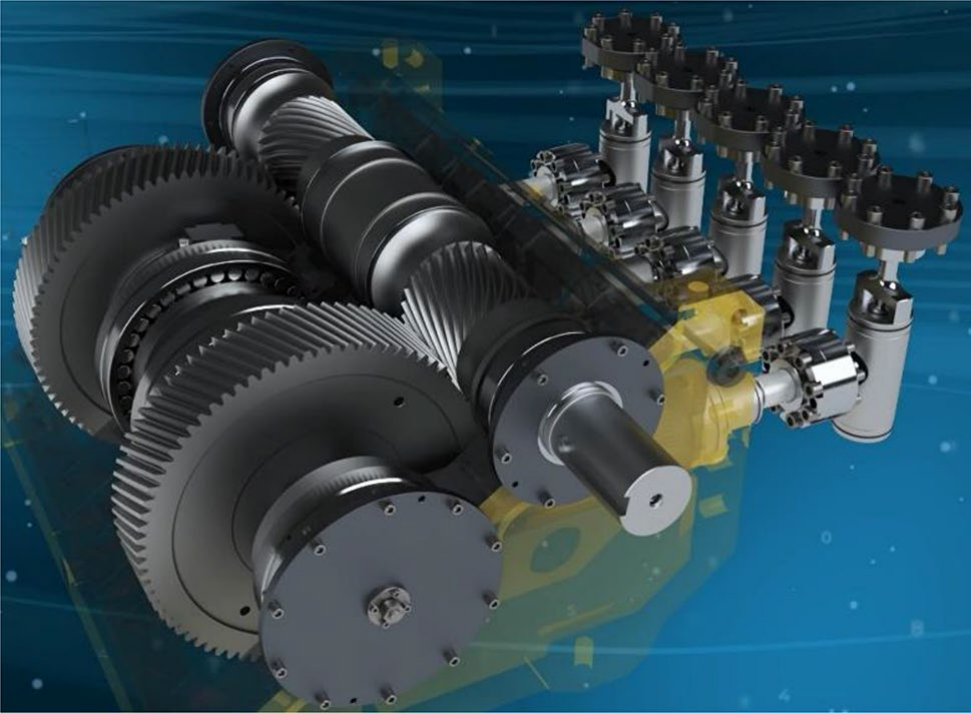
Internal structure of transmission end of emulsion pump.

The emulsion pump studied in this paper is a horizontal five-plunger reciprocating pump. The overall structure is mainly composed of the power end, hydraulic end, control station, pipeline, and base. The drive end provides power for the pump suction and discharge liquid, and its internal structure is shown in Fig. 6. When the mechanism is working, the torque input by the motor drives the gear shaft to rotate, and the gear shaft transmits the torque to the crankshaft through the gear. At the same time, the crankshaft drives the connecting rod to rotate and converts it into a slider reciprocating linear motion.

A three-dimensional model of the power end was established in CREO 5.0.0.0, and the model was meshed using HyperMesh 12.0. Tetrahedral elements were used at the positions of the crankshaft, connecting rod, pin, and slider.

#### Meshing

Because the slideway needs to be analyzed emphatically, hexahedral elements with higher accuracy were used, and the total number of grids was 393,396. Among them, the element sizes of the crankshaft, slideway, slider and connecting rod are 0.015, 0.004, 0.005 and 0.015 m. In order to save calculation time, the crankshaft and connecting rod are modelled coarser to reduce the total number of elements, the grid of the slideway and the slider are appropriately refined, and the more accurate hexahedral element is used on the sleeve.

**Figure 7:**
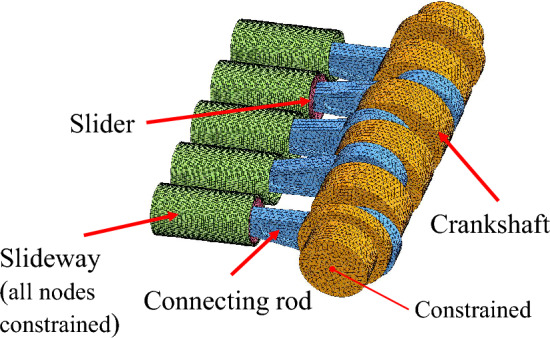
Finite element model of power end.

**Figure 8:**
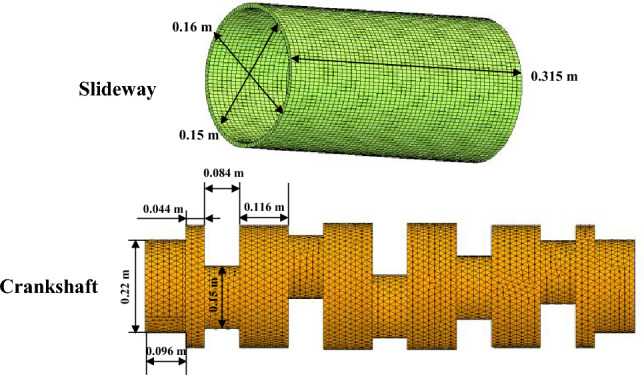
Part dimensions of slide and crankshaft.

The LS-DYNA R10.1.0 program is used for numerical simulation, which is a very famous nonlinear dynamic program. The finite element model and part dimensions of slide and crankshaft are shown in Figs. 7 and 8.

#### Boundary condition

As shown in Fig. 7, the degree of freedom of the linear motion of the center point on the two end surfaces of the crankshaft is constrained, and only the rotational degree of freedom is retained to ensure the stability of the crankshaft motion. The nodes on the outermost circle of the slideway are fully constrained. The elements on the outer circle of the crankshaft are set as rigid elements, and the internal flexible elements are actuated by applying angular velocity to these rigid elements. The speed of the crankshaft is 50 rad/s.

#### Contact

There are four contacts in the model, namely crankshaft and connecting rod, connecting rod and pin shaft, pin shaft and slider, and slider and slideway. The contact mode between the parts is *AUTOMATIC_SURFACE_TO_SURFACE. The friction coefficient between the slider and the slideway in different material combinations is determined by the average value of the friction coefficient dynamic curve in the experiments.

### Materials

The *MAT_PLASTIC_KINEMATIC is used to define the material model of each component, The model is an ideal elastoplastic model. Without considering the influence of strain rate, only density, elastic modulus, Poisson’s ratio and yield stress need to be input into the model. Except for the slider and the slideway, the materials of the other parts are all HT250. Slideway and slider use a variety of different material combinations. All the material model parameters are shown in Table 2.

**Table 2:** Main mechanical parameters of all materials.

Materials	Elastic modulus (N m^−2^)	Poisson ratio	Hardness (HB)	Density (kg m^−3^)	Yield stress (Pa)
HT250	1.38E+11	0.156	170–230	7.28 E+03	
QT400	1.61 E+11	0.274	170–230	7.01 E+03	2.50 E+08
QT500	1.62 E+11	0.293	190–260	7.00 E+03	3.20 E+08
QT600	1.69 E+11	0.286	200–270	7.12 E+03	3.70 E+08

### Wear

To analyze wear, the wear coefficient of each contact surface and the hardness of the material are defined in *CONTACT_ADD_WEAR. This keyword links the wear model to the contact interface. Note that to obtain the result file containing wear information, SPR, and MPR in *CONTACT, DT in *DATABASE_BINARY_INTFOR, and s = 0 in the command line need to be set. SPR and MPR are set to 2 to record the force on the contact surface. In LS-DYNA, for the stability of the calculation, the time step is usually determined by the program itself, and the key word for the time step control is *CONTROL_TIMESTEP.

The LS-DYNA program uses Archard’s wear model to represent the relationship between wear volume and some variables. The expression of the formula is:

2 $$w=k\frac{pv}{H},$$

where *w* is the wear volume, *k* is the wear coefficient, *p* is the contact surface pressure, *v* is the relative sliding speed of two points on the contact surface, and *H* is material surface hardness.

This generalized expression allows the wear amount to be calculated in numerical simulation, many scholars use this formula to define the wear amount in the finite element method. The formula includes many parameters affecting wear in the wear coefficient *K*, and the value of *K* becomes the key to the calculation. However, the determination of *K* is very difficult, and wear experiments are usually used to obtain accurate values. In order to simplify the process of determining *K* and accelerate the wear process in the numerical simulation, this paper ignores the difference in density between different materials and determines the wear coefficient under various material combinations by the proportion of wear amount. The wear amount and corresponding wear coefficient under various material combinations are shown in Table 3.

**Table 3:** Wear amount and wear coefficient under different material combinations.

Combinations	Slider	Slide way	Wear amount/g	Load/N	Wear coefficient
1	QT600	QT400	0.00009	100	1.77e−8
2	QT600	QT500	0.00026	100	5.11e−8
3	HT250	HT250	0.00037	100	7.28e−8
4	QT600	HT250	0.00024	100	4.72e−8
5	QT500	QT500	0.00015	100	2.95e−8

## Numerical simulation results

**Figure 9:**
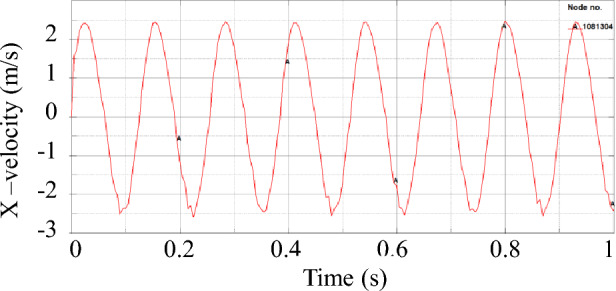
Velocity of slider versus time.

**Figure 10:**
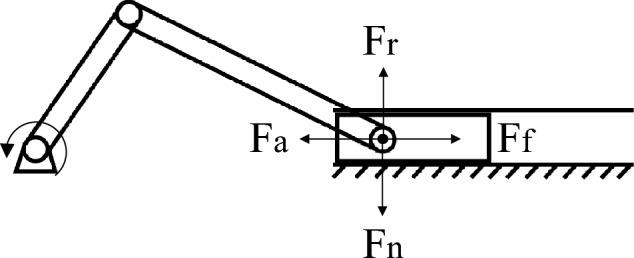
Mechanical model diagram of piston ring cylinder liner system.

**Figure 11:**
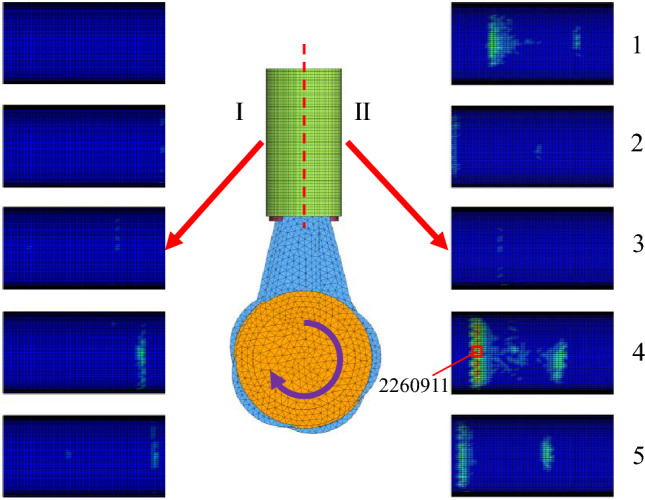
The wear amount on both sides of the slide at a certain moment.

**Figure 12:**
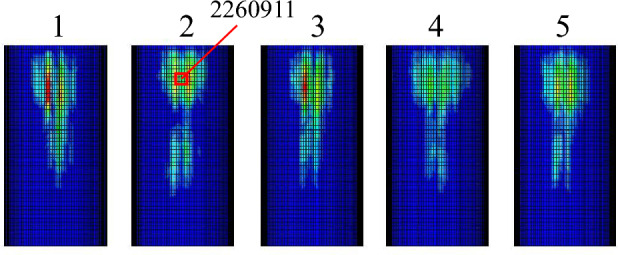
The wear depth cloud map of the slideway at a certain time.

The velocity curve of the slider is shown in Fig. 9, after the circular motion of the crankshaft is transformed into the linear reciprocating motion of the slider, the velocity curve of the slider presents the characteristics of the sine function. The mechanical model of the power end is shown in Fig. 10, *F*_r_ is the vertical component of the force of the connecting rod on the slider, *F*_a_ is the horizontal component of the force of the connecting rod on the slider, *F*_n_ is the reaction force of the slider to the slider, and *F*_f_ is the friction force of the slider from the slider. Figure 11 shows the cloud diagram of the wear depth of the slideway at a certain moment, in the reciprocating motion of the slider, the force of the connecting rod on the slider always has a component force on the side II. Therefore, the wear conditions on both sides of the slideway are different, and the wear amount on the side II is greater than that on the side I. Figure 12 shows the wear depth distribution of the side II of the slideway, it can be seen from the figure that because the position of the pin shaft is not located in the middle of the slider, the contact force between the edge of the two ends of the slider and the slideway is not uniform. The side with high pressure has a wider wear area and deeper wear depth. The edge of the slider near the pin shaft will cause serious wear to the slide.

**Figure 13:**
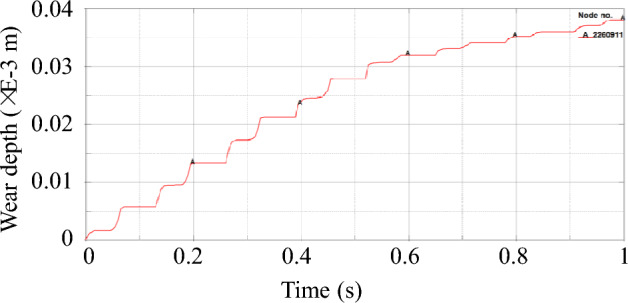
The wear depth curve of node 2260911 on the slideway.

**Figure 14:**
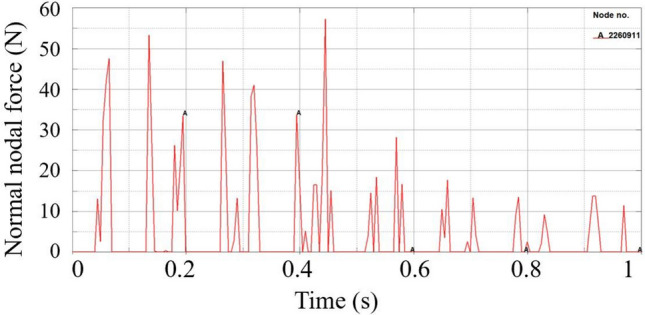
Node normal force of node 2260911.

Figure 13 is the wear depth change curve of node 2,260,911 on the slideway, because node 2260911 on the slideway always contacts the slider during the operation of the power end, and the wear data recorded at this node is complete. The curve shows the characteristics of stepped rise. Each increase in wear depth corresponds to each time the slider passes through the node on the slideway. It can be found from the figure that during the operation of the power end, the wear depth first rises rapidly, and then the slope of the curve gradually decreases. Because the slider passes through this node twice when the crankshaft rotates one circle, the peak of the normal force of the node always appears in pairs from the second peak in Fig. 14. The first peak is the contact force generated when the slider moves away from the crankshaft. From the overall point of view, the former peak is greater than the latter peak. From the wear depth curve, it can also be seen that when the slider moves away from the crankshaft, the increase in the wear depth is greater than the increase in the wear depth when the slider moves towards the crankshaft. The wear of the slipway mainly occurs in the start-up stage. When the slideway is just started, the motion state of the slider inside the slideway is not stable, and the normal force of the node is large. With the increase of the running time, the running state of the slider inside the slideway gradually becomes stable, the contact force of the node decreases significantly, the growth rate of the wear depth also slows down, and the slope of the curve decreases.

**Figure 15:**
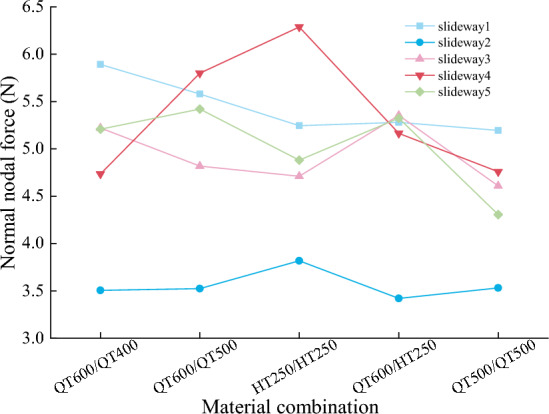
Node normal force.

**Figure 16:**
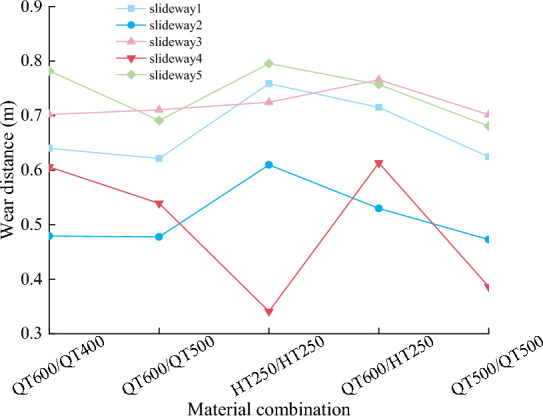
Node wear distance.

**Figure 17:**
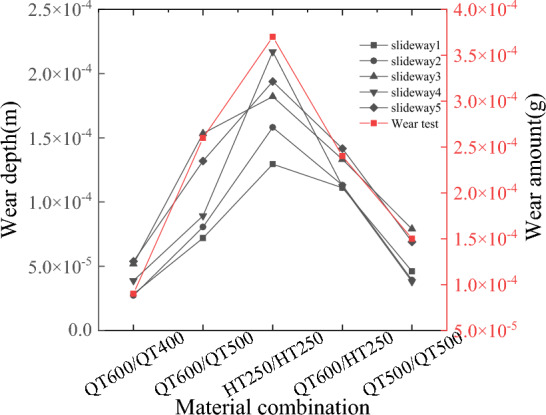
Wear depth and wear amount.

The analysis of slideway wear mainly collects the data of node normal force, wear distance, and wear depth, extracts the data of eight adjacent nodes on the side with larger wear amount, and calculates the average value. Figures 15 and 16 shows the node normal force, wear distance in each material combination. Make the wear depth represents the wear amount, it can be seen from Fig. 17 that the changing trend of the wear quality of each group of experiments is very consistent with the changing trend of the wear depth in Fig. 5, which proves that the finite element model based on Archard’s wear model in LS-DYNA established in this paper can simulate the friction and wear behavior of the friction pair under real conditions.

## Influence parameter analysis

After verifying the simulation method, the model is prepared to simulate more working conditions on the power end, these simulations are often difficult and costly through experiments. The speed of the crankshaft and the clearance between the slider and the slideway are important factors affecting the wear of the power end, because the speed changes the relative motion speed in the Archard’s wear model, and the clearance changes the possible range of the contact force between the slider and the slideway. In this section, the influence of rotational speed and clearance on the wear condition of the slideway is analyzed by parametric adjustment of the model.

### Effect of rotational speed on wear

In the process of emulsion pump design, in order to change the size of the flow, the scheme often adopted is to change the size of the speed. In order to study the wear behavior of the slide at different speeds, the combination of the slider material QT600 and the slide material QT400 was selected to carry out the study of the influence of the speed on the wear behavior.

**Figure 18:**
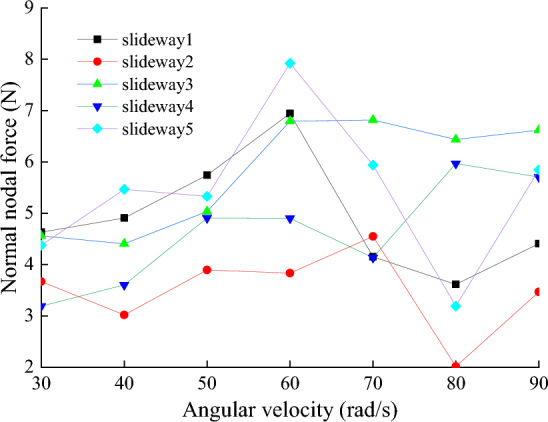
Node normal forces at different rotational speeds.

**Figure 19:**
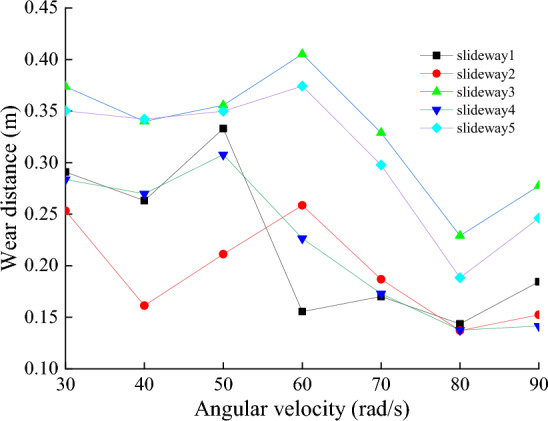
Node wear distance at different rotational speeds.

**Figure 20:**
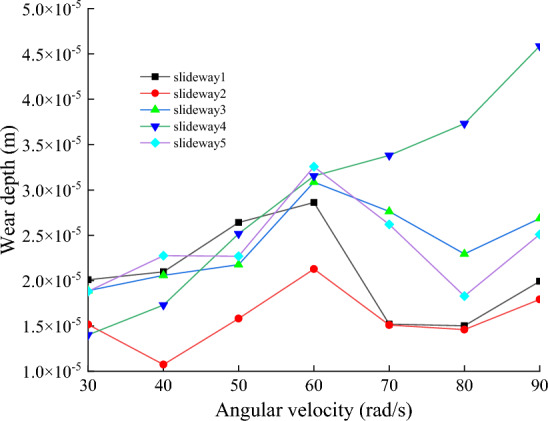
The depth of node wear at different rotational speeds.

Figure 18 shows the average values of the normal nodal force, wear distance and wear depth of the eight nodes on the slide at speeds of 30, 40, 50, 60, 70, 80, and 90 rad/s, respectively. The rotational speed is an important parameter to analyze wear, according to the definition of wear in Archard’s wear model, the faster the relative motion speed is, the greater the wear amount should be. However, the model assumes that the friction pair always maintains a contact state during the movement, and the two surfaces are not separated. During the operation of the power end, the clearance between the slideway and the slider causes the nodes on the slideway not always to be worn, the wear process has a random characteristic. With the increase of the rotational speed, the change of the vibration state of the slider does not have a significant effect on the wear distance at the nodes on the slideway. It can be seen from Fig. 19 that the wear distance does not change significantly when the rotational speed increases from 30 to 60 rad/s. However, the increase of the rotational speed leads to the increase of the inertia force of the slider, the impact between the slideway and the slider becomes more intense, the contact force on the friction pair increases, Fig. 20 shows the wear depth increases with the increase of the rotational speed. It may be that when the speed is lower than 60 rad/s, the increase of contact force is the main reason affecting the wear depth. When the speed exceeds 60 rad/s, the collision between the slideway and the slider is more intense, and the distance of continuous friction between the two is reduced, resulting in a downward trend in the wear distance. This is contrary to the intuitive belief that the faster the speed, the deeper the wear depth.

### Effect of clearance on wear

Due to the influence of factors such as processing and assembly, there may be differences in the size of the clearance between the slider and the slideway. The clearance has a great influence on the dynamic response of the mechanism, it will cause the dynamic response of the mechanism to be different from the ideal situation, which will lead to a more severe collision between the kinematic pair elements and aggravate the dynamic effect of the system. This section studies five different sizes of clearances, which are set between the slider and the cylinder liner.

**Figure 21:**
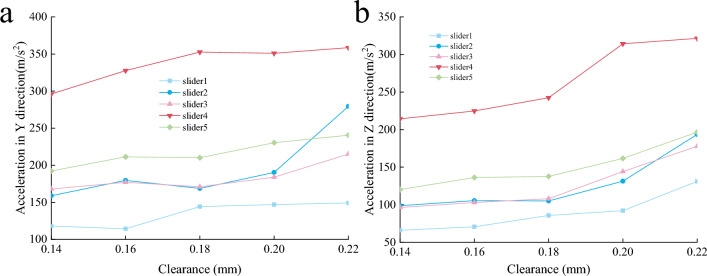
Acceleration in Y and Z directions of slider axis. (**a**) Y. (**b**) Z.

The acceleration dynamic change curve of the slider axis is extracted and the average value of the absolute value is calculated. Figure 21 is the acceleration change of the slider axis in the Y and Z directions with different clearances. The results show that the clearance has a great influence on the acceleration response of the slider. With the increase of the clearance, the acceleration fluctuation is more and more severe, and the oscillation peak is greater. This is because the larger gap causes the impact between the slider and the slide to be more intense, and the stability of the mechanism operation becomes worse, causing a large sudden change in the slider acceleration.

**Figure 22:**
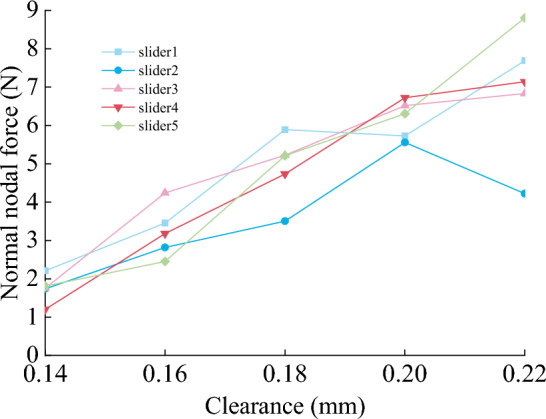
Normal force of nodes under different clearances.

**Figure 23:**
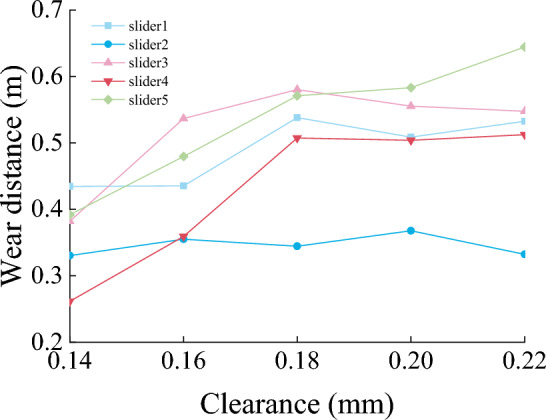
Node wear distance under different clearances.

**Figure 24:**
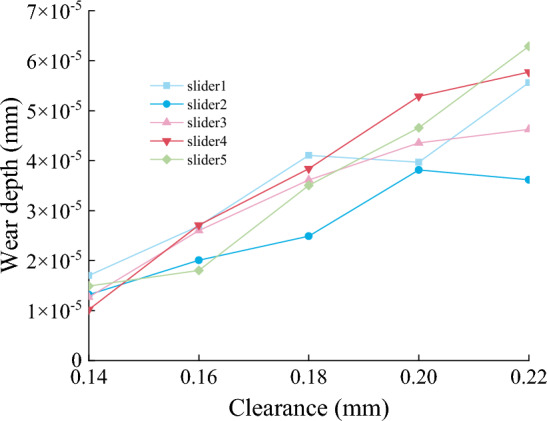
The wear depth of nodes under different clearances.

Figures 22, 23 and 24 show the normal force, wear distance, and wear depth of the eight points on the slideway. From the simulation results, it can be seen that the clearance size has a great influence on the wear amount, as the clearance increases, the wear amount of the node becomes larger, because the larger the clearance, the more severe the collision between the slider and the slideway during the operation of the mechanism, and the greater the collision force. It can be seen from the figure that the normal force of the node increases with the increase of the clearance, and the wear distance increases slowly at first and then has no obvious change, it shows that the change of contact force plays a leading role in the process of clearance change.

## Conclusion

In conclusion, this study successfully employed the finite element method to investigate the friction and wear behavior of the transmission end of an emulsion pump. The wear coefficient determination based on fretting friction and wear experiments, along with the wear depth calculation using Archard’s wear model, demonstrated the ability of LS-DYNA to predict friction and wear behavior accurately.

Additionally, this study quantitatively analyzed the effects of clearance and rotational speed on wear behavior. It was observed that the continuous increase in rotational speed did not necessarily result in more severe wear behavior. Instead, the wear depth initially increased and then decreased with increasing rotational speed. Conversely, the presence of clearance had a significant impact on wear behavior, with larger clearances leading to deeper wear depths. Therefore, it is recommended to maintain a smaller fit clearance during the processing and assembly of the pump.

The findings of this research have practical implications for the selection of chute material and the design of essential equipment parameters, providing valuable guidance for enhancing the performance and longevity of emulsion pumps. The conclusions drawn in this study are applicable to the transmission end of the lubricant pump, but it is not clear whether it is applicable to other types of pumps or mechanical systems. Further research needs to consider other types of pumps or mechanical systems and verify the universality of these conclusions.

## Data Availability

All data generated or analysed during this study are included in this published article.
